# Abortive Treatment of Continued Fever by the Sulphate of Quinia

**Published:** 1853-01

**Authors:** 


*Abortive Treatment of Continued Fever by the Sulphate of Quinia.*—The attention of medical practitioners has lately been called, by communications emanating from regions widely separated from each other, to the efficacy of the sulphate of quinia, administered in large doses, with a view to arresting Continued Fever. The subject is one which appeals so directly and exclusively to experimental research as the mode of developing facts by which its merits are to be determined, that to enter upon an extended discussion of it, irrespective of the data to be furnished by observation, would be, to say the least, a waste of time. Our intention is simply to bring the subject before the minds of our readers, and to suggest some points to be considered in subjecting the plan proposed to the test of experience.

The first of the points just alluded to is, the importance of discriminating *Remitting* from *Continued* fever in making experimental observations of the abortive power of the remedy in question. The bearing of this point is obvious. Quinia does exert, it is now admitted by all, more or less potency in arresting remitting fever, and the question to be settled by the results of observation is, whether the remedy is equally, or measurably applicable to continued fever. That the two forms of fever, common continued or typhoid, and remitting, are often confounded in districts where both prevail, is undoubtedly true. Remitting fever may exhibit in its career many of the features which are more distinctive of typhoid, and hence the discrimination is not always to be made without careful examination, and a thorough knowledge of the various circumstances which are peculiar to the natural history of each. Moreover, the terms *continued* and *typhoid* are frequently employed very loosely, so as to embrace cases of periodical fever in which periodicity is not a prominent feature, and in which the typhoid condition is present.

The importance of this point has been lately impressed upon us by reading an elaborate and able article on the use of quinine in continued fever from the pen of our distinguished and much esteemed friend, Dr. Fenner, of New Orleans. The article referred to is contained in the last No. of the New Orleans Med. and Surg. Journal.

Dr. Fenner, in that article, quotes from a paper of Dr. Thomas Fearn, communicated for the second volume of the *Southern Medical Reports*, in which the efficacy of large doses of quinia in arresting continued fever is claimed to have been exemplified in a striking manner. To borrow Dr. Fenner's language in introducing the quotation: "In this paper Dr. Fearn gives a graphic account of a terrible *continued fever* that prevailed in the year 1831 among a few families residing in the vicinity of Huntsville, (Alabama,) and corresponding very accurately with the descriptions given of the *typhoid*

*fever* at this time prevailing through the Southern States. That fever proved intractable to all the various plans of treatment then in vogue; such as blood-letting, emetics, cathartics, mercurial ptyalism, stimulants, anodynes, and the steam, a Thomsonian course. They *all failed*, and death was slowly but surely sweeping off every one attacked. In this painful and trying emergency, which appeared to defy all the known resources of our art, Dr. Fearn, with the boldness which ever characterises true genius, resolved to try a desperate experiment with the *great febrifuge the sulphate of quinine*. It was determined, in consultation with two other able and experienced practitioners, that in the case of a lady who had been sick three days with the prevailing fever, *when the most perceptible remission should occur*,* the sulphate of quinine should be given in doses of 20 grains, repeated every hour *pro re nata*. *As the remission was expected to occur about midnight*,† Dr. F. remained to superintend the prescription."

Dr. Fenner then proceeds to quote from Dr. Fearn's paper, as follows:— "When the fever was at its highest *there was slight delirium*, *great distress about the precordia*, *and tenderness on pressure*, with *rapid small pulse*, and hot skin. *When it began to abate*,‡ the dose agreed upon was administered. No very sensible effect was observed, and at the expiration of an hour the dose was repeated. Before the expiration of the second hour the pulse was reduced in frequency, and was softer than it had been since she was taken ill. The skin for the first time was moist, *and she was more composed;* nevertheless the third dose was given at the end of the second hour. Shortly after she became tranquil, and fell into a sweet sleep — perspired freely — *the pulse became reduced from* 120 to 80 in the minute, and from that time she was convalescent. Her brother was treated in the same manner, a few days after, and with the same success."

Dr. Fenner adds: "These are the only cases specially reported by Dr. Fearn," etc.

Now, in view of the data afforded by the foregoing very brief account, is the reader warranted in drawing the conclusion that the cases so successfully treated were cases of *typhoid* fever? In answering this question we are compelled to differ from the opinion entertained by our friend, Dr. Fenner. In the first place, there is entire absence of any *positive* evidences of the typhoid character of the disease. Dr. Fearn does not give us even one of the numerous distinctive traits which belong to the natural history of *Continued*, as distinguished from *Remitting* fever. That he is sincere in the

* We have italicized this passage. † Our italics. ‡ All the italics in this extract are ours.

opinion that he was dealing with the former of these two forms of fever we do not doubt, but as respects the grounds on which that opinion is based, we are left without information. Not only is there absence of positive proof, but the facts which are stated in the extract given seem to us to render the conclusion almost certain that the disease was *Remitting* fever. The passages which we have italicized are those which appear to us to sustain this conclusion. On referring to these passages the reader will find evidence of the following features which are highly distinctive of *Remitting* as contrasted with *Typhoid* fever: 1. An abrupt attack. The patient, already presenting great febrile movement, had been ill but three days. 2. Marked remissions, the characteristic giving the name to Remitting fever, and which very rarely occurs in the course of Typhoid. 3. An intensity of febrile movement seldom observed so early in the career of Typhoid, but often in remitting fever. 4. Delirium as early as the third day, which is common in Remitting, and comparatively rare in Typhoid. 5. Distress and tenderness in the precordia denoting gastric irritability — an element of Remitting fever not often observed in Typhoid. Couple the presence of these striking diagnostic criteria of Remitting fever with the absence of any of the criteria of Typhoid, and the evidence contained in the report is all in favor of the former.

If we are correct in this criticism, we have here an illustration of the point under remark, viz., the importance of being careful to discriminate between Remitting and Continued fever, in bringing the abortive efficacy of quinia to the experimental test.

In the article by Dr. Fenner, from which the foregoing extract from Dr. Fearn's report is taken, the writer comments very justly on the few observations made by us on the abortive treatment of Continued Fever by Quinia, contained in our Clinical Reports. We freely admit that the number of cases is too few, and the quantity of the remedy too small, to authorize any deductions as to the merits of the plan of treatment.

Another point to be borne in mind in conducting experimental observations, is the liability to deception by the apparent success of the treatment in cases in which the disease ceases from its own limitations. All who have studied the disease at the bedside are aware that cases not infrequently present themselves in which Continued fever appears to be formed, but ceases after a duration of two, three, or four days. Such cases are classed under the head of *Febricula.* It is by no means always easy to foresee that cases will run this brief, imperfect career; in other words, to discriminate between *febricula,* and true *Continued fever,* prior to the cessation of the fever. There are several circumstances which have an important bearing on this discrimination, but they are not altogether reliable, and, hence, the most experienced and skillful often mistake a case of febricula for continued fever. Practitioners who do not appreciate that the fever in these cases is either a distinct type, or, possibly, instances in which an abortion takes place irrespective of art, not infrequently flatter themselves that the particular measures which they may have employed at the time the febrile movement ceases proved successful in cutting short the disease. Febricula is sometimes observed to prevail at particular seasons, or places, as the common form of fever. This is stated by Prof. Bennett to have been noticed at Edinburgh in the early part of the winter of 1841–2. It is obvious that the cessation of fever, after the administration of quinia, or any other remedy, in isolated, or a limited series of cases, does not establish the efficacy of the abortive treatment. Either the cases of apparent arrest must be numerous enough to outweigh the probabilities of their having been febricula; or, as suggested by Prof. Bennett, "it ought to be a *sine qua non* in all such trials not to commence the treatment until the seventh day." With respect to this suggestion by Prof. B., it may be said with propriety, that it is not improbable an earlier resort to the abortive treatment might be successful, in cases in which it proves inefficacious so late as the seventh day. A sufficiently large proportion of cases in which the treatment was tried earlier, and especially if the trials were repeated at different periods and places, would, therefore, be a fairer method, and equally satisfactory.

The success of quinia in arresting Continued fever has lately been advocated by Dr. Dundas, of Liverpool, Eng. Dr. Dundas' method is to give the remedy in doses of ten grains, repeated every two hours, until five or six doses are taken. Prescribed in this way he declares that the disease may be arrested as certainly as if it were an intermittent, and this statement he affirms to be based on abundant observations by himself and his friends at Liverpool. We have not seen Dr. Dundas' work, and we are not informed concerning the amount, or kind of data which he brings forward to sustain the practice recommended by him. It will not tend to diminish the scrutiny with which the reader should examine whatever facts Dr. D. may have contributed, to know that this author contends for the identity of Continued and periodical fevers.

The test, however, of the plan proposed by Dr. Dundas, (which is essentially the same as that advocated by Dr. Fenner,) is the results of clinical experience. Prof. Bennet was led by the views of Dr. D. to make trial of the practice in a series of cases at the Royal Infirmary of Edinburgh, during the winter session of 1851 – 2, but with a negative result, so far as concerns the interruption of the febrile career, in every case. The cases in which the remedy was tried by Prof. B. were, (so far as our recollection serves,) about fifteen. The cases are reported in full in the Monthly Jour. of Med. Science, Nos. for April and May, 1852. We are not able, at the place where we are writing, to refer to these numbers, or we should give the facts with more precision, as well as more in detail.

Cases of Continued fever are numerous enough in certain districts to determine, without much delay, whether the disease be unaffected by quinia, or not; and it is to be hoped observations will be multiplied sufficiently to put the question at rest. The bare possibility that the advocates of this practice may have grounds for their enthusiasm, should induce observers to make trial of it, especially since no evil results need be anticipated provided the remedy be withheld so soon as any unpleasant symptoms follow a repetition of the doses.

We may remark, in conclusion, that the plan of endeavoring to arrest Continued fever by quinia, is not a novelty. It has been urged, years ago, by practitioners in this country and abroad. Without taking pains, at this time, to make bibliographical researches on the subject, we find in our *scrap book* the following notice of the conclusions of a French observer, M. Boucher, taken from Ranking's Abstract, 1846:

"M. Boucher has undertaken a series of investigations on the physiological and therapeutical action of the sulphate of quinine in large doses in typhoid fever, a subject which has recently attracted much attention among French practitioners. The following are the conclusions which he thinks himself justified in forming, as the result of his own personal observations:

'1. The non-acid sulphate of quinine, in the dose of from two to four *grammes* (40 to 80 grains) in a mixture of 125 grammes, administered in spoonfulls by the mouth, every two hours, or more, does not produce any serious consequences.

'2. It is generally taken with repugnance; often immediately after having been admitted into the stomach producing a temporary nausea, and sometimes vomiting.

'3. The mucous membrane of the digestive passages does not experience from it any injurious influence; there is only some slight sensation of heat in the course of the œsophagus to the cardia.

'4. The eruption of the lenticular spots of the skin and sudamina is not modified, and it appears to be the same also with the intestinal eruption.

'5. Its administration is often followed by a remarkable amendment, which is sometimes only transitory.

'6. The apparent convalescence is generally rapid, but it is not the same with the confirmed convalescence.

'7. This apparent convalescence is owing to the modification af the general condition, the intestines not partaking in this modification.

'8. The nervous phenomena and the slowness of the circulation which are caused by the quinine soon cease when the administration of the medicine is suspended.

'9. It diminishes the headache and often causes it to disappear; the pain is then replaced by heaviness of the head.

'10. It often hastens the return of natural sleep.

'11. Finally, it does not appear that the sulphate of quinine should constitute a special method of treatment, but it may prove serviceable, combined with other means.'"*

Arrest of the disease does not appear among the effects of the remedy, which was administered in large doses, and the results apparently studied with care in a large collection of observations.

* Credited by Ranking to Gaz. Med. and Prov. Med. Journ., Jan. 1846.

